# Drunkenness and Its Treatment in the Sixteenth Century

**Published:** 1905-06-03

**Authors:** 


### DRUNKENNESS AND ITS TREATMENT IN THE SIXTEENTH CENTURY.

One would hardly expect a dissertation on drunkenness and its treatment from a German doctor in the sixteenth century. But Dr. Christopher Wirtzung devotes a chapter to "The Detestable Sicknesse Drunkennesse" in his Generall Practise of Phisicke, first published in 1568 and translated into English in 1617. He says: "It will perchance offend the gentlemen drunkards that I have here put their sickness of drunkenness after the infectious plague and agues, and that I have compared their sickness to those. But I will omit here the loss of everlasting life, disdaine of humanity and credit, consumption of worldly wealth, strife, murder, blasphemy of God, disclosing of secrets and many more ungodly works that ensue thereof: wherefore we will only here treat of the hurt that the body may hereby take thereof, and let others judge afterwards whether this sickness be not worse than the ague, yea, worse than the plague itself. For Solomon speaketh not in vain that drunkenness hindreth all wisdom, which cannot be otherwise confirmed than that it maketh a man a fool: darkeneth it not the understanding, enfeebleth it not the brains, the memory and all the senses, bringeth it not more forgetfulness with it than is perceived in young children. Behold only how the hands, the feet, the head, yea, the whole body tremble and quake, how the sight is darkened, the tongue stammereth, and how there is not one member of the body that is able to discharge his duty aright. Yea, say they, this sickness hath neither need of the physician's nor apothecary's counsel, make thereof as weighty a sickness as you can, it may be holpen with a very pleasant medicine, that is, sweet sleep. Whereto I say and answer: Oh, good drunkard, there followeth with the time some other thing thereof, to wit, that thereby the good complexion of your liver is spoiled, so that it doth not engender any good blood but other bad humours whereby the Dropsy afterwards ensueth . . . . whereof is caused fearfulness, frightings, speaking in the sleep, heavy dreams, the loss of all good colour, and lastly a madness itself, item the palsy, lethargy, the falling sickness, and diverse such cold diseases. Is there not provoked through this drunkenness a corruption of the stomach . . . . the gout, and to conclude, an untimely death. Whereof to helpe this extreme sickness there is no certainer or speedier remedy than Sobrietas and fasting or abstinence and albeit that this receipt seems to be altogether bitter, sour, and unpleasant for these Aleknights, yet notwithstanding it will expel the forsaid malady.

"Whereas some have so weak a stomach that they are of necessity constrained to drink wine, notwithstanding they are of so feeble a brain that how soberly soever they drink it it disturbeth their heads and maketh them drunken. For this purpose may these things following be used: to wit, juice of quinces or raw quinces well chewed and the juice taken down; likewise the juice of citrons and lemons, or of their syrups to hinder drunkenness. Honey is also much commended if it be taken after much drinking of wine, for thereby will the vapours of the wine be driven downwards that it cannot weaken the understanding nor the brains. Bitter almonds confected, conserves of gilliflowers after that you have drunken much to prevent drunkenness. In old times men did make a garland of saffron flowers and did wear it on their heads; the same virtue is ascribed to the blue violets, and it is said that white coleworts cut into salads and the same eaten or the seeds thereof chewed in the mouth, should hinder drunkenness. The precious stone the amethyst should also preserve men from drunkenness. But for to defend and prevent all contagions that proceed of drunkenness we will add something more. First, he must be urged to vomit with oxymel of squills, with radish seed, with a feather dipped in oil and put into the throat and thereupon he must fast and sleep: and after he awaketh again you must froth (foment) his members to wit, his hands and feet with warm water wherein is sodden roses, camomile, and a little salt, and tempered with some oil of violets whereby the vapours may be drawn downwards. And lay a cloth upon the head that is made wet with juice of cucumbers, purslaine (porcellana) or in any of their waters: the patient must keep himself herein very sober, and use light meats, coleworts, lentils, young pullets and young pigeons dressed with the juice of lemons and pomegranates; the prepared and confected coriander is also very good, and sleeping thereupon until all the wine be digested. It chanceth, oftentimes, that drunken folks have great thirst, which, if it cometh not through the abundance of wine, then give fair fountain water to drink, and sour fruits to eat. And it is a common opinion that watered wine doth sooner infect the head than pure wine. But this is to be understood of gross and thick wine, which, by the putting to of a little water, is the more subtiller, whereby the vapour sooner fumeth into the head."

Nothing is now known of Dr. Christopher Wirtzung, who wrote this, except that he was born at Augsburg in 1500, practised in Heidelberg, and died in 1571.

